# Soil minerals affect taxon-specific bacterial growth

**DOI:** 10.1038/s41396-021-01162-y

**Published:** 2021-12-20

**Authors:** Brianna K. Finley, Rebecca L. Mau, Michaela Hayer, Bram W. Stone, Ember M. Morrissey, Benjamin J. Koch, Craig Rasmussen, Paul Dijkstra, Egbert Schwartz, Bruce A. Hungate

**Affiliations:** 1 Department of Biological Sciences, Northern Arizona University, Flagstaff, AZ 86011 USA; 2 Center for Ecosystem Science and Society, Northern Arizona University, Flagstaff, AZ 86011 USA; 3 Earth and Biological Sciences Directorate, Pacific Northwest National Laboratory, Richland, WA 99354 USA; 4 Division of Plant and Soil Sciences, West Virginia University, Morgantown, WV 26506 USA; 5 Department of Environmental Science, University of Arizona, Tucson, AZ 85721 USA; 6 Present Address: Department of Ecology and Evolutionary Biology, University of California, Irvine, CA 92697 USA

**Keywords:** Biogeochemistry, Microbial ecology, Biogeochemistry

## Abstract

Secondary minerals (clays and metal oxides) are important components of the soil matrix. Clay minerals affect soil carbon persistence and cycling, and they also select for distinct microbial communities. Here we show that soil mineral assemblages—particularly short-range order minerals—affect both bacterial community composition and taxon-specific growth. Three soils with different parent material and presence of short-range order minerals were collected from ecosystems with similar vegetation and climate. These three soils were provided with ^18^O-labeled water and incubated with or without artificial root exudates or pine needle litter. Quantitative stable isotope probing was used to determine taxon-specific growth. We found that the growth of bacteria varied among soils of different mineral assemblages but found the trend of growth suppression in the presence of short-range order minerals. Relative growth of bacteria declined with increasing concentration of short-range order minerals between 25–36% of taxa present in all soils. Carbon addition in the form of plant litter or root exudates weakly affected relative growth of taxa (*p* = 0.09) compared to the soil type (*p* < 0.01). However, both exudate and litter carbon stimulated growth for at least 34% of families in the soils with the most and least short-range order minerals. In the intermediate short-range order soil, fresh carbon reduced growth for more bacterial families than were stimulated. These results highlight how bacterial-mineral-substrate interactions are critical to soil organic carbon processing, and how growth variation in bacterial taxa in these interactions may contribute to soil carbon persistence and loss.

## Introduction

Soil microbial activity is responsible for one of the largest fluxes in the terrestrial carbon (C) cycle. Yet, this activity comes from an immensely diverse collection of co-existing organisms from all branches of the tree of life, with a single gram of soil often containing more than a billion organisms and thousands of species [1]. This biological diversity arises in part from physical and chemical complexity of the soil environment which includes a diverse soil mineral matrix.

The diverse geologic histories and parent materials of Earth’s terrestrial landscapes give rise to the variable mineral composition of soils. Different soil mineral assemblages have been found to favor specific microbial community development through their surface characteristics such as charge and surface area, nutrient content, and stage of weathering [2–7]. Soil minerals can also influence microbial communities by affecting pore size [8, 9], particle size [10, 11], aggregate formation [12, 13], and rates of microbial processing of SOC [14]. Particularly, clay minerals have reactive surfaces that affect many soil properties, including nutrient accessibility, soil moisture, and the accumulation and persistence of soil organic carbon (SOC) [15]. Bacteria predominately inhabit microaggregates and the clay fraction [13, 16–19], where they are thought to live mostly attached to surfaces as individual cells or in small colonies or biofilms [20–22]. Clay particles can protect bacteria from predation and unfavorable climatic conditions [2], yet can limit growth by restricting microbial mobility [23] as well as access to organic matter (OM) [8].

Fresh OM from vegetation can be stabilized via occlusion within aggregates, adsorption to mineral surfaces, and complexation with metals [21, 24, 25]. Mineral-associated bacteria frequently live on surfaces with little accessible soil OM [22]. Consequently, these microorganisms may depend on fresh OM that comes into proximity with bacterial cells via diffusion through pores [13, 26]. Low-molecular weight organic substrates are more readily consumed by bacteria than high molecular weight compounds, which require extracellular enzyme production to break down to a form that can be assimilated through microbial membranes [24]. Abundant and phylogenetically clustered bacterial taxa were found to increase in relative abundance after addition of the chemically simple substrates glycine and sucrose [27]. In contrast, fewer and phylogenetically dispersed taxa increased in relative abundance in response to polymers such as cellulose, lignin, and tannins [27]. Thus, which bacterial taxa respond to fresh organic carbon may depend not only on the mineral composition and diversity of the soil, especially clay minerals, [28] but also on the chemical composition of fresh organic carbon entering the soil.

The soil mineral assemblage strongly influences SOC dynamics, especially short-range order (SRO) minerals [29–32]. These non- to poorly-crystalline clay-sized particles—such as allophane, imogolite, and ferrihydrite—protect newly introduced OM or decomposition products from microbial consumption [31, 33]. The presence of SRO minerals has been suggested to control both SOC content as well as mean residence time [29], so may exert considerable control over the quantity and availability of SOC to microbial communities.

Understanding how minerals and microorganisms influence the availability of a wide spectrum of organic compounds in soil may help predict activities of bacterial taxa and the stabilization of soil OM. While much information has accumulated about the presence/absence and relative abundances of individual microbial taxa [6, 27, 34], methodological constraints have made it challenging to observe phenotypes of individual microbial taxa, such as growth rates, in situ.

In this study, we used quantitative stable isotope probing (qSIP) with ^18^O-labeled water to measure the assimilation of ^18^O from water into newly synthesized DNA of soil bacteria, an approach that makes it possible to estimate taxon-specific growth in microbial communities, in situ [35–37]. To evaluate how the soil mineral assemblage affects growth, we quantified bacterial taxon-specific growth in soils from three different parent materials from the same western North American mixed-conifer forested ecosystem type. We also evaluated how the addition and chemical form of carbon modulated the effect of soil minerals on growth, and the roles of taxonomic identity in predicting bacterial growth in soils with different mineral assemblages.

## Materials and methods

### Site

Three soils were collected from the A horizon (0–11 cm) in June of 2012 from sites in the California Sierra Nevada mountains with similar mixed conifer vegetation, mean annual temperature, slope, and clay content as described previously [38, 39]. The primary difference among sites was the parent material of the soils: andesite, basalt, or granite, which from this point forward we refer to the three soils by their parent material [14, 33]. The andesite soil had the highest proportion of SRO minerals (quantified as the sum of allophane and iron-oxyhydroxides), the basalt was intermediate, and the granite had low amounts of SRO minerals (Table 1). The granite soil instead was dominated by hydroxy-interlayered vermiculite and kaolinite. The SRO mineral content of these soils positively correlated with total SOC content. The pH varied from moderately acidic (pH 5.8 in the andesite and 6.0 in the granite) to slightly acidic (pH 6.5 in the basalt). For more information on soil chemical and mineral properties, see [Table S1] [38, 39]. At each of the three sites, five field replicates were collected 15 m apart, sieved to 2 mm, and composited together as one field sample per site. Thus, our design does not consider variation within sites, but we preserve the inter-soil comparison. Soils were stored at 4 °C to reduce microbial activity prior to the incubation.

**Table 1:** Soil taxonomic classification [38, 39], physicochemical parameters, as well as mean CO_2_ respired (µg CO_2_–C g^−1^ soil) from each C addition treatment (water-only, root exudate, and litter C) after seven days of incubation.

Parent material	Soil taxonomic classification	Soil C properties		Microbial biomass		SRO minerals		CO_2_-Total (µg CO_2_–C g^−1^)		
		SOC (g kg^−1^)	Soil *C*:*N*	C (µg g^−1^)	*C*:*N*	Fe_o_ (g kg^−1^)	Allophane (g kg^−1^)	Water	Exudate	Litter
Andesite	Mesic humic haploxerand	98.5 ± 9.4	24.4 ± 0.8	786.8 ± 24.13	10.5 ± 1.3	6.8 ± 0.4	78.0	56.5^a^ ± 4.1	187.5^c^ ± 1.1	96.9^b^ ± 2.7
Basalt	Mesic typic haploxerept	60.0 ± 2.1	19.6 ± 0.2	170.5 ± 6.81	4.6 ± 0.4	2.7 ± 0.2	50.0	48.6^a^ ± 2.9	192.3^c^ ± 2.5	99.2^b^ ± 1.5
Granite	Mesic humic dystroxerept	31.1 ± 1.7	28.0 ± 0.3	570.8 ± 16.18	13.7 ± 2.3	2.8 ± 0.3	nd	201.6^c^ ± 0.5	345.2^e^ ± 6.8	251.2^d^ ± 11.8

Mean respiration values (*n* = 4) followed by different superscripted lowercase letters (a–e) are significantly different from each other at *p* < 0.05 (Tukey’s HSD post hoc test). Total SRO mineral content of each soil (g kg^−1^ soil) was calculated as the sum of oxalate-extractable iron (Feo) and allophane.

### Incubation

In July of 2013, soil samples were weighed to 1 g dry weight, placed into separate 15 mL plastic Falcon tubes, and rewetted to 60% of field capacity at room temperature for five days to allow for acclimation after ~1 year of refrigeration. Prior to the incubation, soils were air dried for 24 h to reduce soil moisture content.

At the incubation’s onset, 200 µL of water was added to all treatments: 97 atom% ^18^O-water for the labeled treatments and natural abundance deionized water to the parallel unlabeled treatments. Treatments included the three soils (andesite, basalt, and granite), three C-addition treatments (water-only, root exudates, and ground pine litter), and two isotope treatments (H_2_^18^O at natural abundance and at 97 atom %) with four replicates for each treatment, yielding 144 microcosms. For both the root exudate and litter additions, 350 µg C g^−1^ soil dry weight equivalent (at a concentration of 80 mg C mL^−1^) was added to each sample at the beginning of the incubation. Expressed as relative molar abundances, the root exudate mixture consisted of commercially available: four parts carbohydrates (fructose, glucose, sucrose, and lactate), two parts organic acids (succinate, malate, and citrate) to one part amino acids (serine, cysteine, and alanine) [Sigma-Aldrich Co. LLC], [38]. The litter consisted of dried, ground *Pinus ponderosa* needles [14]. A parallel incubation was conducted separately on 40 g soil to measure CO_2_–C respired from the different soils and C addition treatments published previously [38]. Further information on the litter and exudates used are in Table S2. During the 7-day incubation, tubes were opened on days 2 and 5 to re-aerate the soils, such that median [O_2_] declined only slightly, from 21 to 20.3% before re-aeration (90th percentile [O_2_] declined to 19.2%; O_2_ concentrations were estimated assuming 1:1 stoichiometry of CO_2_ production to O_2_ consumption). Samples were harvested after 1 week and stored at −40 °C until DNA extraction. DNA was extracted from 0.5 ± 0.01 g soil (dry weight equivalent) using a FastDNA Spin Kit for Soil (MP Biomedicals) and stored at −40 °C.

### Density centrifugation and fractionation

DNA was separated by density centrifugation by adding 800–1000 ng DNA to a solution containing 2.55 mL saturated (1.9 g mL^−1^) cesium chloride, 450 µL gradient buffer (200 mM trisaminomethane, pH 8, 200 mM potassium chloride, 2 mM Ethylenediaminetetraacetic acid), and ~200 µL of TE buffer in 3.3 mL OptiSeal ultracentrifuge tubes (Beckman Coulter). Ultracentrifuge tubes were balanced in a Beckman TLN-100 rotor and spun at 127,000 × *g* for 96 h using an OptimaMAX TL ultracentrifuge.

The resulting cesium chloride gradient was fractionated into approximately 14, 150–200 µL fractions. Fraction density was measured using a digital refractometer (Reichert), then purified by using an isopropanol precipitation method and re-suspended in 50 µL TE buffer. DNA concentrations in each fraction were measured with the Qubit BR dsDNA assay (Invitrogen).

### qPCR

Quantitative PCR was conducted on all DNA fractions to measure 16S rRNA bacterial gene copy numbers. Standard curves were made using tenfold serial dilutions of 16S rRNA gene copies extracted from soil and amplified using bacterial 16S qPCR F515/R806 primers [40]. The 10 µL triplicate reactions contained 1 µL of template DNA added to 9 µL reaction mixtures of 0.75 µM of each primer, 0.01 U µL^-1^ Phusion Hot Start II Polymerase (Thermo Fisher Scientific), 1× Phusion HF buffer (Thermo Fisher Scientific), 1.5 mM MgCl_2_, 1× EvaGreen, 6% glycerol, and 200 µmol L^−1^ dNTPs. Each qPCR assay was performed using a protocol of 95^o^C for 1 min followed by 40 cycles of 95 °C for 30 s, 64.5 °C for 30 s, and 72 °C for 1 min on an Eppendorf Mastercycler ep Realplex system (Eppendorf). For each assay, 16S rRNA gene copy numbers were determined using a regression equation to relate the cycle threshold value to the known gene copy numbers of the standard curve. All standard curve efficiencies were above 90% and *R*^*2*^ values were above 0.995 (Table S3).

### Sequencing

We sequenced all fractions that fell between densities of 1.66–1.74 g mL^−1^, producing a range of 7–13 fractions sequenced per ultracentrifuge tube. The V3–V4 hypervariable region of the 16S rRNA gene was amplified in triplicate from each fraction with a 10 µL reaction mixture containing 1 µL of genomic DNA, 5 µL Phusion high-fidelity PCR master mix with HF buffer (New England BioLabs, Inc.), 0.75 µL dimethyl sulfoxide (DMSO), 1.75 µL molecular-grade water, and 1 µM of each forward (5′-GTGCCAGCMGCCGCGGTAA-3′) and reverse (5′- GGACTACVSGGGTATCTAAT-3′) primers. Initial PCR reaction products were pooled, checked on a 1% agarose gel, 10-fold diluted, and used as template in the subsequent tailing reaction with region-specific primers that included the Illumina flowcell adapter sequences and a 12 nucleotide Golay barcode (15 cycles identical to initial amplification conditions). Products of the tailing reaction were purified with carboxylated SeraMag Speed Beads (Sigma-Aldrich) at a 1:1 v/v ratio described previously [41] and quantified by Picogreen fluorescence. Equal concentrations of the reaction products were then pooled; the library was again bead-purified (1:1 ratio), quantified by qPCR using the Library Quantification Kit for Illumina (Kapa Biosciences), and loaded at 11 pM (including a 30% PhiX control) onto a MiSeq instrument (Illumina Inc.) using a v2 2 × 150 paired-end read chemistry. Sequence data from this study are available in the NCBI short-read archive under accession number PRJNA701328.

### Data analysis

The resulting DNA sequence data of forward and reverse reads (FASTQ) were demultiplexed using QIIME 2 release 2021.4 [42] and denoised using the q2-DADA2 pipeline [43]. Denoised sequences were clustered into amplicon sequence variants (ASVs) at 100% sequence identity and taxonomy was assigned using the q2-feature-classifier, classify-sklearn naïve Bayes taxonomy classifier against the SILVA 138 database [44]. Samples were removed if they had less than 3000 sequence reads, and ASVs were removed if present in less than three samples. This filtering resulted in 11,320 unique prokaryotic ASVs at a frequency of 13,699,617 reads (>99% read retention). We estimated the excess atom fraction (EAF) ^18^O enrichment for each ASV as a proxy for growth using calculations described previously [35]. Taxon-specific weighted average density was calculated within the natural abundance H_2_^18^O and 97 atom % enriched H_2_^18^O parallel treatments from the distribution of 16S rRNA gene copies of each ASV in the CsCl density gradient. The shift in weighted average density from the unenriched to enriched samples was used to calculate the proportion of isotope incorporation using a model of oxygen isotope substitution in DNA. We removed ASVs from the analysis if they were not present in at least three fractions within a sample or at least two replicates within a treatment, leaving 3476 prokaryotic ASVs. Due to sequencing quality filtering and sample loss, the granite-litter treatment had two replicates; other treatments had four replicates. An effect of ultracentrifuge tube on weighted average density was found, likely due to slight differences in CsCl density within each tube, so was corrected as described previously [45]. As detection of archaeal ASVs was negligible in these soils, we focused our analyses on bacterial ASVs. We calculated EAF ^18^O for each ASV using R version 4.1.1 [46] and data.table [47], and scripts associated with qSIP calculations publicly available at https://www.github.com/bramstone/qsip.

A PerMANOVA test [“adonis” function; 48] was used with 1000 permutations on the Euclidean distances between group centroids of samples of both ASV relative abundance and EAF ^18^O to assess the effects of soil and substrate on community structure and growth, followed by visualization using non-metric multidimensional scaling (NMDS).

To quantify the proportion of variance in EAF ^18^O explained by soil type and taxonomy across the different substrate amendments, we used restricted maximum likelihood and variance partitioning analysis on shared ASVs, with soil type and fresh organic carbon substrate addition nested within taxonomy [49]. Shared ASVs were included if they were present in all soil types and belonged to a phylum with at least two orders and a family with at least three members present to reduce misattribution of variance on taxonomy. Through this selection, 310 ASVs classified into 45 families, 37 orders, 13 classes, and seven phyla were included in the analysis. The “lme” function [50] was used to run the restricted maximum likelihood analysis, then the “varcomp” function [51] was used to determine variance partitioning using the following model specification: *EAF* ~ 1 + 1|*Phylum/Class/Order/Family/Genus/ASV/soil/substrate*.

To estimate the mean relative growth across all shared ASVs within each community, we calculated the mean EAF ^18^O for each separate sample, averaged across each treatment. We then conducted a two-way analysis of variance (ANOVA) followed by post-hoc Tukey’s HSD contrasts between soil and substrate type to determine if mean relative growth across shared ASVs between treatments were significantly different (*α* = 0.05).

The mean difference and 95% confidence interval (CI) in growth between soil types and substrate C treatments was quantified by bootstrapping the difference at the family level (1000 iterations with replacement). We used the 95% bootstrapped CI of the difference of means as a threshold for statistical interpretation. To visualize growth of the 310 different ASVs that were screened above, a heatmap was constructed using the “superheat” package [52] depicting the mean relative growth (EAF ^18^O) for each of the 45 families across the three soils and substrate amendments and sorted by phylum.

At the ASV level, “SRO stimulated taxa” were identified if the slope of the relationship between EAF ^18^O and the proportion of SRO minerals was significantly positive (*α* = 0.05). “SRO-unaffected” ASVs had a slope of EAF ^18^O by proportion of SRO mineral that was not significantly different from zero, while “SRO suppressed taxa” were identified as ASVs that had a significantly negative slope in growth under increasing soil SRO content.

## Results

### Soil respiration

The andesite and basalt soils, the two soils abundant in SRO minerals, despite higher total SOC content, had lower respiration rates after one week of incubation compared to the granite soil (Table 1). Under water-only conditions, the granite soil community respired about four times more CO_2_–C as the andesite and basalt soils (Table 1). Fresh C substrate increased total CO_2_–C respired from all soils, with exudate addition eliciting greater CO_2_–C respired than litter addition.

### Bacterial community composition

**Fig. 1:**
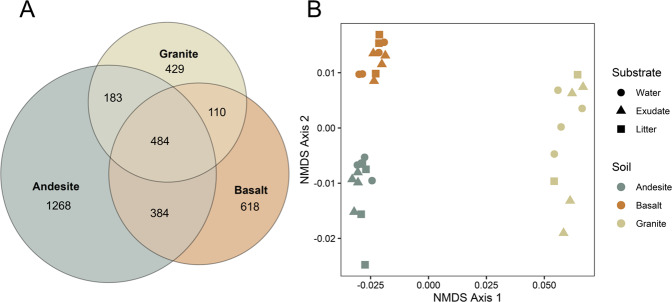
Variation in bacterial community composition of the three soils studied. **A** Venn diagram of the number of shared and unique bacterial amplicon sequence variances (ASVs) within each soil. **B** Non-metric multidimensional scaling (NMDS) of soil communities at the ASV level under water-only conditions, as well as under exudate and litter C addition. Distances of points within NMDS are based on relative abundances of ASVs.

Of the three soil types, the andesite had the greatest bacterial richness with 2319 ASVs present in all three C addition treatments, the basalt had 1596 ASVs, and the granite soils had 1206 ASVs (Fig. 1a). Out of 3476 ASVs, 484 (13.9% of all detected ASVs) were present in all soils and C addition treatments. The andesite soil had the highest measured 16S rRNA gene copy abundance with 2.52^6^ copies g^−1^ soil, with lower abundances in the basalt (1.15^6^ copies g^−1^ soil) and granite (1.06^6^ copies g^−1^ soil; Fig. S1a). The 484 ASVs present in all soils comprised more than 57% of the andesite and basalts’ communities and more than 80% of the granite’s community under water-only conditions.

The soils significantly differed in bacterial community composition (PerMANOVA *F*_2,28_ = 39.02, *R*^2^ = 0.71, *p* > 0.01; Fig. 1b), and substrate C addition did not affect changes to community composition (PerMANOVA *F*_2,28_ = 1.65, *R*^2^ = 0.03, *p* = 0.13). The most abundant bacterial phyla across the soils were Actinobacteria and Proteobacteria, together comprising 59% of the andesite, 54% of the basalt, and 72% of the granite communities in the control treatments (Fig. S1b). Acidobacteria were also abundant across the soils, with lower abundances of Bacteroidota, Gemmatimonadota, Chloroflexi, Firmicutes, Myxococcota, Planctomycetota, and Verrucomicrobia.

### Bacterial growth

**Fig. 2:**
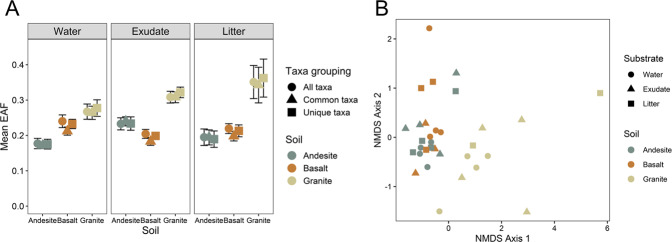
Bacterial community growth responses in soils of varying SRO mineral content under different substrate-additions. **A** Grand mean excess atom fraction (EAF) ^18^O of all bacterial amplicon sequence variants (ASVs) present within each soil, common taxa shared across all soil types (484 ASVs), as well as taxa unique to each soil type. Error bars represent standard error of the mean. **B** Non-metric multidimensional scaling (NMDS) plots of EAF ^18^O for the 484 common taxa across soils and substrate-addition treatments (stress level = 0.11). Distances of points within NMDS are based on mean EAF ^18^O of ASVs.

Soil, not substrate type, significantly affected the mean relative growth (EAF ^18^O) of taxa within each soil. We quantified the mean EAF ^18^O averaged across all ASVs present, shared ASVs across soils, and unique ASVs within each soil to determine if taxa unique to a soil on average had different relative growth rates to taxa shared across soils and did not find a significant difference between these different ASV groupings within each carbon amendment treatment (Fig. 2a). The average relative enrichment was 0.1 EAF ^18^O greater across treatments in the granite soil (Fig. 2a, *p* < 0.01, Tukey’s HSD) compared to the andesite and basalt, which did not differ from each other. Substrate largely did not affect mean relative growth across taxa for all, shared, and unique taxa, except for an interaction between the basalt soil decreasing in EAF ^18^O under exudate addition compared to water-only conditions (Table S2, *p* < 0.05).

Soil type significantly affected relative growth of bacterial ASVs shared in all treatments while addition of C weakly affected relative growth (Fig. 2b). Across all soils and substrate additions, soil type explained about four times more variation (dispersion) in bacterial community EAF ^18^O than addition of either exudate or litter C for shared taxa (Fig. 2b; PERMANOVA, Soil *F*_2,29_ = 7.90 *R*^2^ = 0.34, *p* < 0.01; Substrate *F*_2,29_ = 1.69 *R*^2^ = 0.04, *p* = 0.09).

For most taxa present in all three soils, the highest growth response occurred in the granite across substrate amendments. Here, between 6 and 25 times as many ASVs had higher relative growth in the granite soil (difference in means at 95% CI) compared to the andesite and basalt soils across treatments. However, the granite soil had the lowest ASV richness, so in terms of absolute number of growing taxa under water-only conditions, there were 40% and 25% fewer total ASVs growing in the granite compared to the andesite and basalt, respectively (Table 2).

**Table 2:** Bacterial ASV richness of entire communities, as well as the number of growing ASVs within each community (EAF ^18^O 95% bootstrapped confidence intervals not overlapping zero, and mean EAF ^18^O for each ASV greater than 0.05).

Soil	Substrate	ASV richness	Number of growing ASVs	Number of ASVs growing more than in:		
				Andesite	Basalt	Granite
Andesite	Water-only	2939	2522	–	31	18
	Exudate	3327	2833	–	87	20
	Litter	3196	2729	–	53	33
Basalt	Water-only	2349	2040	70	–	27
	Exudate	3080	2674	9	–	10
	Litter	2037	1787	48	–	27
Granite	Water-only	1710	1525	184	173	–
	Exudate	1755	1521	116	242	–
	Litter	1519	1384	291	27	–

Number of ASVs growing more for each soil (row by column) were estimated by the 95% CI difference in means, out of the 484 common taxa present in all soils.

Soil type explained much of the proportion of explained variation in relative growth compared to taxonomy and substrate type. From restricted maximum likelihood analysis and variance components estimation, taxonomy accounted for 37.7% of the explained variation in EAF ^18^O values, soil accounted for 53.5%, and substrate type accounted for 8.8% (Fig. S2). Of taxonomy, phylum accounted for 6.3% of the total explained variation, and lower taxonomic levels (family to ASV) explained the rest (31.4%).

**Fig. 3:**
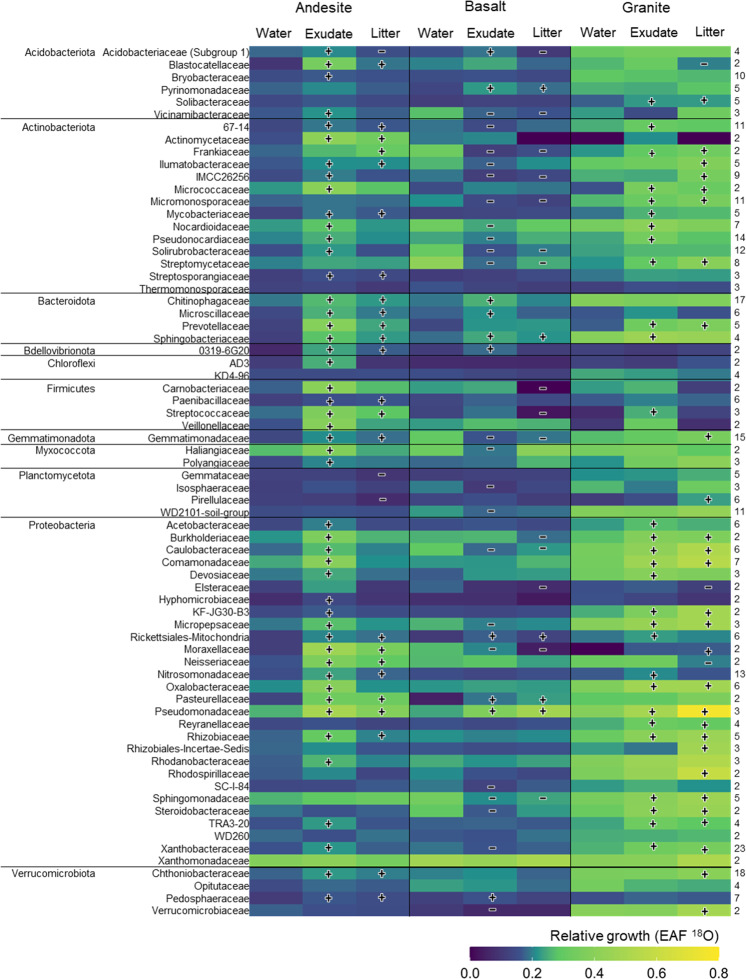
Growth responses by family in different soils and substrate additions. Heatmap of mean relative growth rate (excess atom fraction ^18^O) of 70 families present in all soils under water-only (control), exudate-addition and litter-addition with at least two member taxa within each family. Darker colors depict lower growth rates, and lighter (green to yellow) depict higher growth rates. Bars with “+” symbols indicate families that had significantly higher growth rates under exudate or litter addition compared to the water-only treatment of the same soil type, while bars with “−” symbols indicate significantly lower growth rates compared to water-only conditions (CI 95%). Families are grouped by phylum alphabetically. Numbers in parentheses to the right of the heatmap indicate the number of ASVs grouped within each family.

At the family level, the type of fresh C affected growth of different taxa, and the effect differed across soils. In the andesite soil, 48 out of 70 families’ growth were stimulated in response to exudate addition compared to water-only conditions—twice as many as due to litter addition—and about half the observed families grew significantly more under exudate that litter addition (bootstrapped difference of means at 95% CI, Fig. 3). In the granite soil, 29 families’ relative growth was stimulated by exudate addition, similar to 28 families under litter addition. Notably, in basalt soils, fresh C led to *reduced* growth of 22 families from exudate addition and 15 families under litter addition compared to water-only conditions. Here, addition of either form of C elicited negative responses from a diverse array of families, particularly within Actinobacteria and Proteobacteria (Fig. 3).

Some families’ growth responses varied more by the type of fresh C addition, others varied more by soil type, and others were not affected by either. For instance, Xanthomonadaceae consistently grew the most averaged across soils and C addition treatments but was not affected by the addition of exudate or litter C (Fig. 3). The only family whose relative growth was stimulated from exudate addition across soils was Sphingobacteriaceae, and Pseudomonadaceae was the only family that consistently increased in growth from either litter or exudate addition regardless of soil type (95% CI difference in means). Other families differed in growth response due to fresh C depending on the soil they inhabited. For instance, Streptomycetaceae grew less under fresh C addition in the basalt, grew more in the granite, and did not change in the andesite. For other families, there appeared to be an interactive effect between soil and fresh C addition, such as the case of Acidobacteraceae (Subgroup 1) increasing in growth in response to exudate C in both the andesite and basalt but decreasing due to litter addition, with substantial relative growth with no change across treatments in the granite soil (EAF ^18^O > 0.31; Fig. 3). Thus, across phyla, families displayed a wide range of growth responses across soils with and without fresh substrate C.

**Fig. 4:**
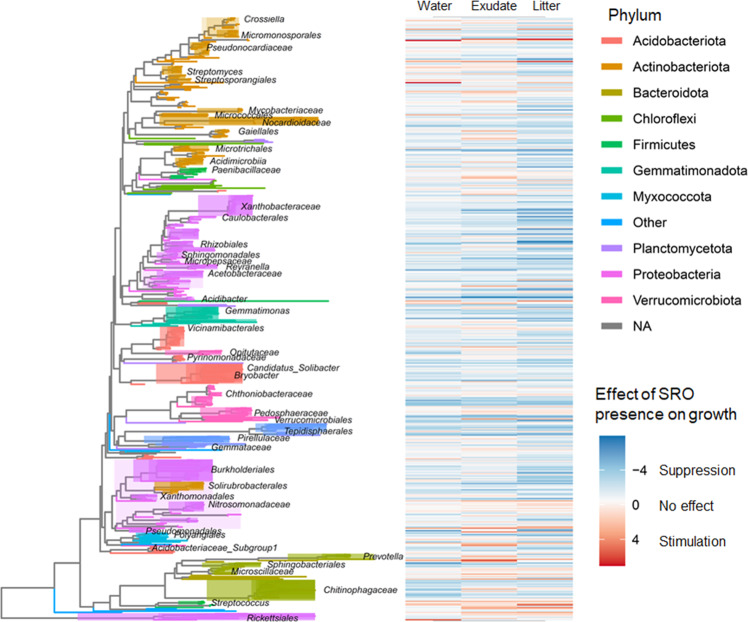
Phylogenetic tree of bacterial taxa based on 16S rRNA gene sequences and associated growth responses in presence of SRO minerals. The phylogenetic tree is colored by phylum and labeled by phylogenetic group. The heatmap depicts the direction and magnitude of the slope of excess atom fraction (EAF) ^18^O per ASV over short-range order (SRO) mineral proportion within soil. Negative slopes correspond to amplicon sequence variants (ASVs) which grew less in soil with greater SRO minerals (indicating SRO suppression on growth) and positive slopes (red) correspond to ASVs which had positive correlation of relative growth rate and SRO mineral content (SRO stimulation on growth).

Of the 484 bacterial ASVs present in all soils, most ASVs’ relative growth were either unaffected or suppressed in the presence of SRO minerals (Fig. 4). Under water-only addition, only three ASVs (less than 1% of ASVs), all of which belonged to the Actinobacteria (in genera *Atopobium, Rothia*, and *Actinomyces*) were stimulated by increasing SRO content, and 35.3% of ASVs were suppressed by SRO presence in terms of relative growth (slope of SRO content by EAF ^18^O significantly negative, *α* = 0.05). Fresh C addition slightly increased the number of positive responders to SROs, with five ASVs under exudate addition and nine ASVs under litter addition having relative growth significantly increase under greater SRO mineral concentration. More ASVs still had suppressed growth with SRO under fresh C addition, with 29.7% of ASVs under exudate addition exhibiting suppressed growth with greater SRO mineral concentration and 35.5% of ASVs under litter addition (Fig. 4). The taxa that had suppressed growth with increasing SRO mineral content comprised 26–48% of 16S copies within each soil community, while those that were stimulated under increasing SRO content comprised less than 0.5% of each soil community under different substrate additions (Table 2).

## Discussion

Our experimental design used a lithosequence approach, where naturally occurring soils were selected based on their parent materials and at sites where other soil-forming factors were held constant. This approach is arguably a weaker foundation for mechanistic inference, for example, compared to a synthetic soil of defined mineral gradients or microcosms of pristine mineral surfaces. However, this approach is useful to infer effects of soil forming factors on biogeochemical processes, and in this case, on microbial growth, in naturally occurring soils and microbial assemblages. Additionally, the use of natural soils allowed us to explore the dynamics of minerals and C on microbial assemblages which at present cannot be fully replicated in situ.

We found that soils with high SRO mineral content have lower bacterial growth across taxa. In general, if a taxon was present in all three soils, its highest relative growth occurred in the granite soil (containing the lowest SRO mineral content), which may have been due to more available OM from weaker organo-mineral interactions [15]. Despite occurring in the soil with the lowest SOC content, bacteria in the granite generally had the highest relative growth. Corresponding to the higher relative growth of bacteria, the granite soil also had the highest rate of CO_2_ production. A lower SOC content has been found to be one of the more important limiting factors on total community bacterial growth across a wide range of soils [53]. However, our results suggest that the relative quantity of SOC content alone does not explain bacterial growth. Mineral-protection of SOC reduces its accessibility for microbial growth, a factor that is increasingly acknowledged as being important for SOC persistence [24]. In addition to having negligible SRO mineral phases, the granite soil is dominated by the phyllosilicate clay minerals kaolinite and hydroxy-interlayered vermiculite. With weaker organo-mineral interactions, these clays may have fostered conditions for faster-growing bacteria. Non-expansible layered silicates such as kaolinite and hydroxy-interlayered vermiculite have been shown to reduce adsorbed OM [54]. The interlaying of vermiculite by hydroxide minerals reduces reactive mineral surface area and exchange capacity, reducing the capacity for interactions between OM and minerals [55].

The lower bacterial relative growth observed in the andesite and basalt soils may have been caused by the higher abundance of SRO mineral phases in those soils. Previous work from these soils found that both SOC content and residence time were primarily explained by SRO mineral content [33]. This suggests that presence and greater concentration of SRO minerals decreased microbial access to SOC, and therefore limited bacterial growth for most taxa. SRO minerals can also inhibit microbial activity through several other mechanisms: sorption of organic compounds to mineral surfaces, promotion of micro-aggregate formation, and Al-toxicity [29, 33], even in the presence of fresh OM [56]. We found that bacteria grew more slowly in SRO-rich soils, in which CO_2_ mineralization rates were also lower. However, under all C addition treatments, a majority (>85%) of bacterial ASVs grew significantly in the andesite and basalt soils across diverse phyla (Table 2). This suggests that while bacterial growth may in general be slower in soils with SRO mineral phases, most bacterial taxa present are still able to grow.

Bacterial ASV richness was greater for the andesite and basalt soils, despite lower average relative growth compared to the granite soil, which may be due to greater number of microsites and niche space associated with high specific surface area of SRO minerals [21]. SRO minerals have strong adsorption capacity leading to slower microbial growth, yet they also have large and highly reactive surface areas with greater potential for microbial attachment [57, 58]. Presence of clay surfaces can be advantageous to soil microorganisms, allowing for greater protection from predation, providing a surface on which to produce biofilms, as well as providing access to nutrients and C sources [4, 6]. The physical and chemical complexity of both the SRO phases, as well as the organo-mineral interactions, may have been the cause of greater bacterial richness [6], yet the strong sorption of SOC on those same SRO phases may have inhibited SOC access to bacteria, leading to slower growth.

Contrary to our expectations, exudate addition did not consistently enhance bacterial growth compared to litter addition for a wide range of ASVs across soil types. This experiment was only seven days, so microbial growth responses from the ground litter input may have largely been due to rapid leaching of dissolved organic compounds from the litter’s soluble fraction (Table S2) [59, 60]. We observed that the influence of C addition depended more on soil mineral composition. This is evidence that the effect of mineral composition in soils may be important not only for the potential for SOM stabilization, but also bacterial community composition and growth potential. Previous work on pristine clay minerals also found a greater influence of mineral type over the effect of simple root exudate C or more complex litter C [61]. However, we did find that more ASVs were detectably growing under exudate addition compared to litter, especially in the andesite soil, and that fewer total ASVs in the basalt and granite soils were growing under litter addition compared to water-only conditions.

In the basalt soil, exudate addition suppressed overall growth (Fig. 2a), and both exudate and litter suppressed growth of numerous families (Fig. 3) compared to water alone, suggesting a complicated relationship between minerals, SOM, and microbial activity. While growth for many bacterial taxa was suppressed, substrate C addition did increase respiration from all three soil communities. Possibly, fungi responded positively to substrate addition in the basalt soil, suppressing responses of bacteria through competition [62]. Future studies could use fungal qSIP to address such interactions. The lower total microbial biomass in the basalt compared to the andesite and granite soils may have limited the basalt bacterial community from accessing and growing on fresh substrate C in part due to greater direct sorption of the fresh C on mineral surfaces, especially the soluble exudate C. The framework posed by Sokol et al. [25], suggests that soils with lower microbial density form mineral-associated OM of fresh plant C via direct sorption, with less initial microbial uptake and processing compared to soils with higher microbial density.

Our finding that bacterial taxonomy accounted for around a third of the explained variation in bacterial growth is consistent with evolutionary history as a strong determinant of bacterial growth in soil [49]. We found that most of the explanatory power arose from classification at the level of family or lower, with little variation explained at higher levels of classification. We also found that the soil parent material—and thus soil mineral assemblage—explained considerable variation in bacterial growth, relatively more than found among soils along an elevation gradient [49]. This also suggests that the growth response of the whole microbial community was more important than growth responses of specific bacterial populations.

Most soil microorganisms live in close proximity to minerals, and these mineral-associated microorganisms play important roles in biogeochemical cycling, soil formation, and providing nutrients to plants. The mineral composition of soil influences the structure of both bacterial and fungal communities [18, 21]. Here, we show evidence that soil mineral composition affects growth of soil bacteria: as SRO mineral content increases, the relative growth of numerous bacterial taxa declines.

## Supplementary information

Supplementary Information

Supplemental Table 3
