# Supplementary material for: Soil minerals affect taxon-specific bacterial growth
Source: ISME J. 2021 Dec 20;16(5):1318–26. doi: 10.1038/s41396-021-01162-y (PMC9038713; doi:10.1038/s41396-021-01162-y)
Supplement: Supplementary file 1 — Supplementary Information [file 41396_2021_1162_MOESM1_ESM.docx]

**Supplementary Information for**

**Soil Minerals Affect Taxon-specific Bacterial Growth Rates**

Authors: Brianna K. Finley^1,2,6^*, Rebecca L. Mau^2^, Michaela Hayer^2^, Bram W. Stone^2,3^, Ember M. Morrissey^4^, Benjamin J. Koch^1,2^, Craig Rasmussen^5^, Paul Dijkstra^2^, Egbert Schwartz^1,2^, and Bruce A. Hungate^1,2^

Affiliations:

*^1^Department of Biological Sciences, Northern Arizona University, Flagstaff, AZ 86011, USA; ^2^Center for Ecosystem Science and Society, Northern Arizona University, Flagstaff, AZ 86011, USA; Earth and Biological Sciences Directorate, Pacific Northwest National Laboratory, Richland, WA 99354, USA;* ^4^*Division of Plant and Soil Sciences, West Virginia University, Morgantown, WV 26506, USA;* *^5^Department of Environmental Science, University of Arizona, Tucson, AZ, USA 85721; ^6^Current affiliation: Department of Ecology and Evolutionary Biology, University of California, Irvine, CA, USA 92697*

*Corresponding author: BK Finley [bkfinley@uci.edu](mailto:bkfinley@uci.edu)

Conflicts of Interest: The authors declare no conflict of interest.

**Table S1.** Soil characterization data (± SE) for granite, basalt, and andesite soils.

|  | Basic soil properties | | | |  | Mineralogy variables (g kg^-1^)* | | | | | | | | | | | | | | |
| --- | --- | --- | --- | --- | --- | --- | --- | --- | --- | --- | --- | --- | --- | --- | --- | --- | --- | --- | --- | --- |
| Parent Material | pH 1:1 H2O | Clay  (g kg-^1^) | CEC (cmol kg^-1^) | Base saturation (%) |  | Fe_d_ | Fe_o_ | | Si_o_ | | Al_o_ | | Al_p_ | | Allophane | | Clay mineralogy | | |  |
| Granite | 6 | 77 | 18 | 46 |  | 4.4 (0.1) | | 2.8 (0.3) | | 1.1 (0.1) | | 6.4 (1.0) | | 2.7 (0.8) | | _ | | HIV > K > >G |  |  |
| Basalt | 6.5 | 63 | 30 | 51 |  | 7.0 (0.4) | | 2.7 (0.2) | | 9.9 (1.7) | | 17.9 (2.1) | | 7.6 (0.6) | | 50 | | SRO >> HIS > K = G |  |  |
| Andesite | 5.8 | 94 | 40 | 51 |  | 18.6 (1.3) | | 6.8 (0.4) | | 11.2 (0.5) | | 31.7 (0.7) | | 10.5 (0.1) | | 78 | | SRO >> G = K |  |  |

*Data is a subset of soil data presented in Rasmussen et al. (2006) and Finley et al. (2018). Mineralogy variables represent average values of three pedons sampled at each field site. Abbreviations are: CEC, cation exchange capacity; C, carbon; MBC, microbial biomass carbon; Fe_d_, sodium dithionite extractable Fe (crystalline Fe) ; Fe_o_, SRO Fe-oxyhydroxide (oxalate-extracted Fe); Si_o_, oxalate-extractable Si; Al_o_, oxalate-extractable Al; Al_p_, pyrophosphate-extractable Al; G, gibbsite; HIS, hydroxy interlayered smectite; HIV, hydroxy interlayered vermiculite; K, kaolinite/halloysite; SRO, short range order. Allophane content estimated from the Al_o_-Al_p_/Si_o_ molar ratio based on Dahlgren (1994). Clay mineralogy was determined by X-ray diffraction and minerals are listed in order of relative abundance based on relative peak intensity in X-ray diffractograms.

**Table S2.** Information on litter and exudate substrate components: percent of total mass, as well as percent carbon, percent nitrogen, and C/N ratio for each substrate component. Litter was separated via ANKOM fractionating (Finley et al. 2018).

| Substrate | Substrate Fraction | Substrate Components | % of total mass |  | %C | %N | C/N |
| --- | --- | --- | --- | --- | --- | --- | --- |
| Litter | Bulk |  |  |  | 48.02±0.1 | 1.48±0.02 | 32.37±0.42 |
|  | NPE | non-polar extracts (fats, oils, soluble cell contents) | 61.8±1.18 |  | 48.69±1 | 1.87±0.07 | 26.03±0.53 |
|  | WS | water soluble hemicellulose | 9.37±0.16 |  | 36.66±6.14 | 0.62±0.1 | 63.83±13.78 |
|  | AS | acid soluble cellulose | 16.88±0.23 |  | 45.01±1.91 | 0.6±0.19 | 120.93±53.14 |
|  | ADL | lignin, recalcitrant materials | 10.84±0.37 |  | 60.92±0.53 | 1.71±0.03 | 35.59±0.6 |
| Exudates | Bulk |  |  |  | 37.47±0.11 | 1.23±0.02 | 30.41±0.5 |
|  | Sugars | fructose, glucose, sucrose, lactate | 70.23 |  | 40.53±0.53 | 0 | - |
|  | organic acids | succinic acid, malic acid, citric acid | 21.31 |  | 38.01±1.42 | 0 | - |
|  | amino acids | serine, cysteine, alanine | 8.46 |  | 34.82±3.1 | 13.53±1.2 | 3.5 |


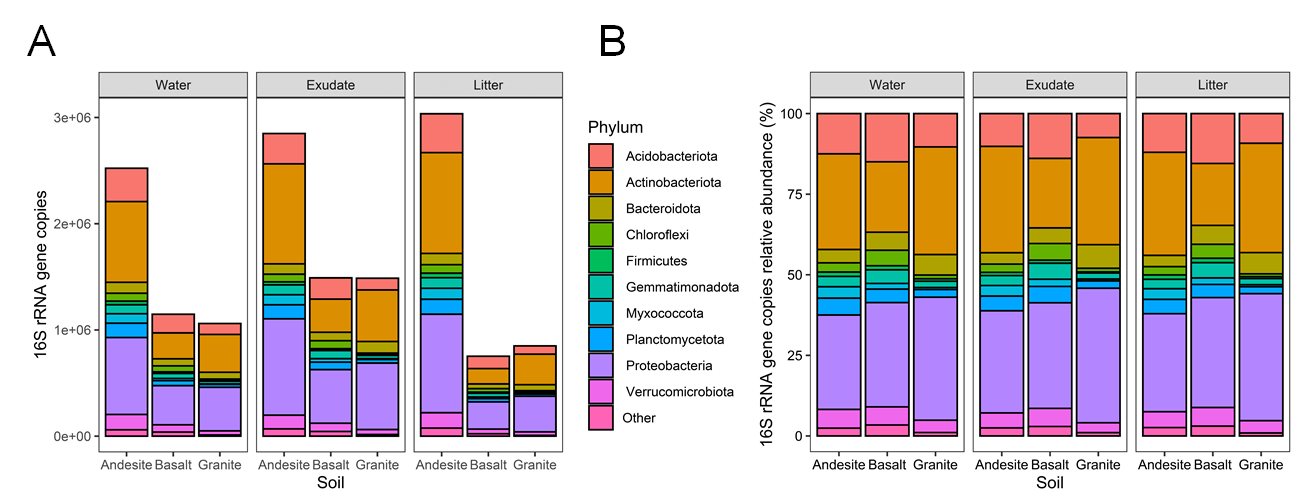


**Figure S1.** A) Total gene copies (16S rRNA) from extracted DNA (mean of 4 replicates) for each soil and substrate C addition treatment. B) Relative abundances of phyla present in soil samples.


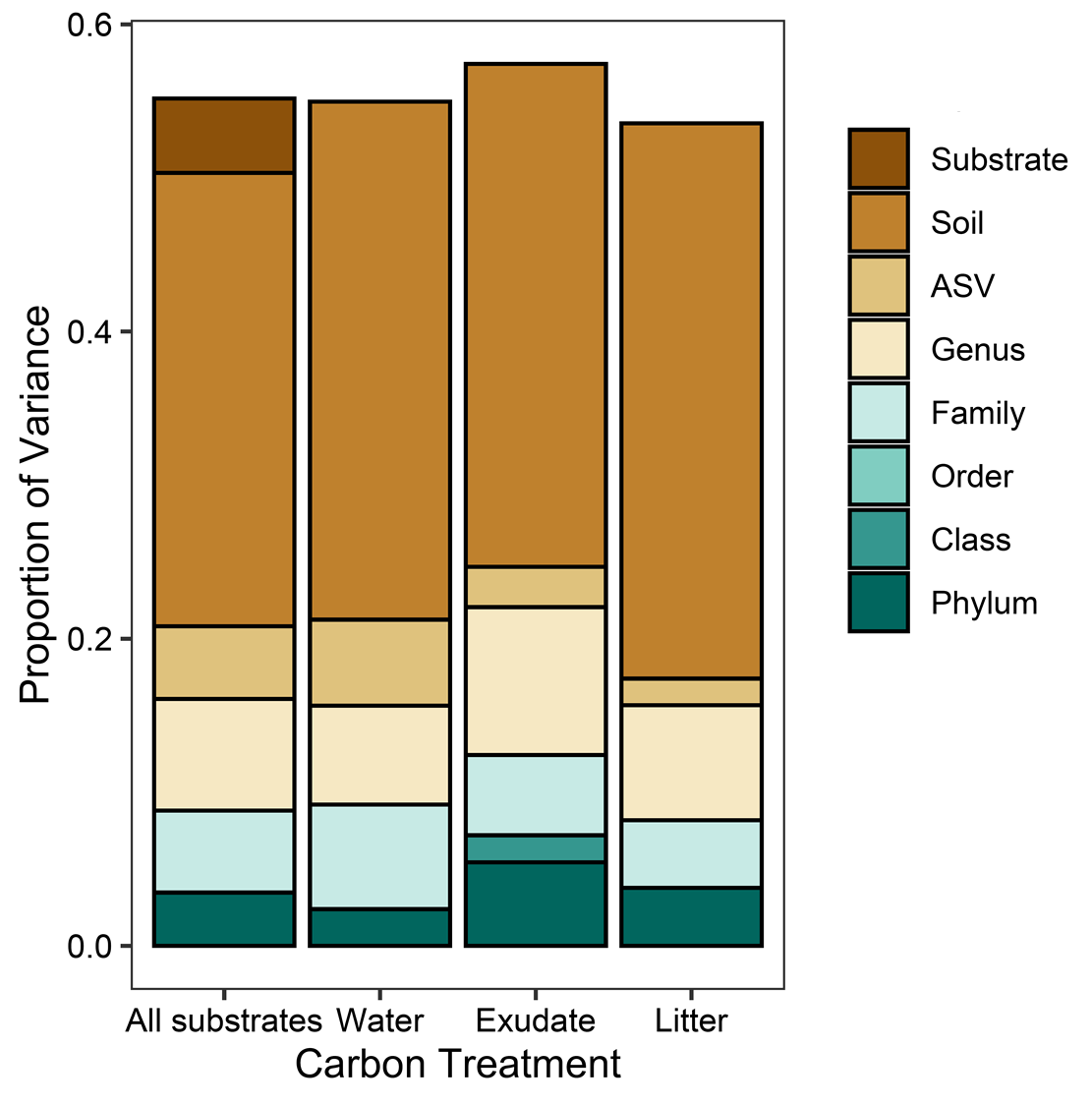


**Figure S2.** Variance in relative growth rate explained by soil type and taxonomy from nested linear models on EAF ^18^O under all C substrate additions. Stacked bars represent proportional variance explained by nested taxonomic levels within soil and substrate. ASVs included in this model were the 310 ASVs present in all soils and substrate additions and passed filtering criteria of at least 2 orders within a phylum and 3 ASVs within a family.
